# High scan-rescan repeatability of AI-enabled coronary plaque quantification from coronary CT angiography

**DOI:** 10.1007/s00330-026-12598-1

**Published:** 2026-05-12

**Authors:** Joel Lenell, Caroline Park, Jacek Kwiecinski, Guadalupe Flores Tomasino, Sevan Letourneau, John D. Friedman, Donghee Han, Elliot. K. Fishman, Shenghan Lai, Daniel S. Berman, Piotr J. Slomka, Damini Dey

**Affiliations:** 1 Biomedical Imaging Research Institute, Departments of Biomedical Sciences and Medicine, Cedars Sinai Medical Center, Los Angeles, CA USA; 2 Department of Interventional Cardiology and Angiology, Institute of Cardiology, Warsaw, Poland; 3 Department of Cardiology, Smidt Heart Institute, Cedars–Sinai Medical Center, Los Angeles, CA USA; 4 Department of Radiology and Radiological Science, John Hopkins Medical Center, Baltimore, MD USA; 5 Department of Epidemiology, John Hopkins Medical Center, Baltimore, MD USA; 6 Division of Artificial Intelligence in Medicine, Department of Medicine, Cedars–Sinai Medical Center, Los Angeles, CA USA

**Keywords:** Coronary artery disease, Computed tomography, Atherosclerosis

## Abstract

**Objectives:**

To evaluate the reproducibility of plaque quantification from serial cardiac computed tomography (CCTA) scans by a systematic side-by-side approach using validated AI-enabled software with both scan-specific and fixed attenuation thresholds.

**Materials and methods:**

Thirty participants from two centers underwent serial CCTA within a short timeframe (median 6 days [IQR 0–30]). Volumes and burden (defined as plaque volume indexed by vessel volume) of total plaque (TP), calcified plaque (CP), non-calcified plaque (NCP), and low-density NCP (LD-NCP) were quantified per-patient while comparing the serial scans side-by-side. Analyses were done by two assessors using consensus readings to reduce individual bias, and inter-scan differences were compared using mean differences and the repeatability coefficient (RC, defined as 1.96 × standard deviation).

**Results:**

Mean age was 59 years (70% male). Median TP volume was 159 mm^3^ (IQR 81–160) with a dominance of NCP, 88 mm^3^ (IQR 42–143). Intraclass correlation coefficients were excellent across all plaque metrics (≥ 0.96). Mean absolute difference between scans was 15.0 mm^3^ for TP volume, 1.5% for TP burden (RC 25.5 mm^3^ and 2.6%). Mean differences for CP, NCP, and LD-NCP volumes were 6.8, 11.1, and 2.3 mm^3^ (RC: 14.8, 21.7, and 5.1 mm^3^), and for burden 0.6%, 1.2%, and 0.3% for CP, NCP, and LD-NCP, respectively (RC: 1.3%, 2.4%, and 0.8%). Scan-specific thresholds presented with lower repeatability coefficients and narrower limits of agreement than fixed thresholds.

**Conclusion:**

Scan-rescan reproducibility was high across all plaque subcomponents for serial plaque quantification using a systematic assessment method and scan-specific attenuation thresholds with AI-enabled plaque software.

**Key Points:**

***Question*** *Can comparisons of serial coronary CT angiography scans side-by-side offer reproducible quantifications of coronary plaque, and do scan-specific attenuation thresholds provide higher consistency than fixed thresholds*?

***Findings*** *AI-enabled plaque quantifications side-by-side were highly reproducible, and scan-specific attenuation thresholds provided higher consistency than fixed thresholds*.

***Clinical relevance*** *This study helps clinicians to reliably assess changes in coronary plaque volumes over time by defining the reproducibility across serial scans. The study also favors scan-specific attenuation thresholds over fixed thresholds for optimal reproducibility in the quantification of noncalcified plaque and calcified plaque*.

**Graphical Abstract:**

**Figure:**
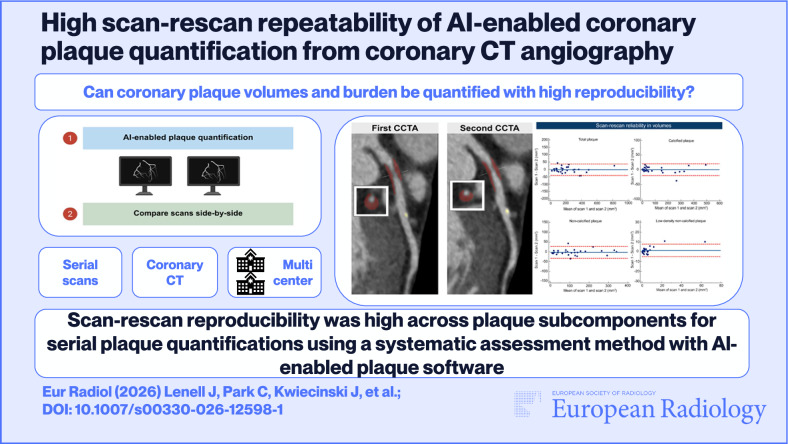

## Introduction

Cardiac computed tomography angiography (CCTA) is the cornerstone in the diagnostic work-up of suspected atherosclerotic coronary artery disease (CAD) due to its high sensitivity in identifying plaque [1]. However, not all plaques are the same. Novel artificial intelligence (AI) enabled software allows for assessment of CAD burden by quantification of the total plaque (TP) volume and its sub-components comprising calcified plaque (CP), non-calcified plaque (NCP), and low-density NCP (LD-NCP) [2].

CPs are considered stable, and the risk of a myocardial infarction (MI) has been reported to decrease with higher volumes of high-density CP (typically defined as CT attenuation ≥ 1000 Hounsfield units [HU]) [3]. Conversely, NCP and particularly LD-NCP have been associated with increased risk of MI [4].

Clinical reporting of qualitative high-risk plaque features is reader dependent, and there is evidence of substantial inter-rater variations [5]. Quantitative assessments of high-risk LD-NCP may be a more reproducible and clinically actionable metric than the qualitative assessments widely practiced today.

Statin treatment and PCSK9 inhibitors have been shown to promote an increase in CP with a simultaneous reduction in NCP [6, 7]. Serial quantitative plaque acquisitions have the potential to help monitor and guide these pharmacotherapeutic treatment interventions by the identification of changes in plaque composition over time. However, the assessment of longitudinal changes in plaque is dependent on the reliability of CCTA plaque measurements.

Reproducibility in blinded quantitative plaque assessments of repeated CCTA scans has been reported to suffer from substantial variability [8, 9]. To tackle this challenge, a recent expert consensus paper suggested a new approach to quantitative serial CCTA acquisitions with assessment of serial scans side-by-side to adjudicate changes in plaque distribution [10].

In this study, we aimed to assess the inherent variability in plaque volume acquisitions between serial scans using the latest generation of AI-enabled software and the proposed side-by-side method. As a secondary objective, we also examined the precision of scan-specific vs pre-defined (fixed) HU-thresholds for plaque adjudication.

## Materials and methods

This retrospective study was carried out in accordance with the Declaration of Helsinki and was approved in its entirety by the institutional review boards at Johns Hopkins University and Cedars–Sinai Medical Center. All participants provided written informed consent.

### Participants

Thirty participants, selected based on available serial CCTA scans at short intervals, were retrospectively included for evaluation of scan-rescan reproducibility of coronary plaque quantifications. Nineteen participants (mean age 52 ± 6 years) had undergone serial scans at Johns Hopkins University within 100 days, median 15 days (interquartile range [IQR] 6–38), and 11 participants (mean age 71 ± 12 years) were scanned at Cedars–Sinai Medical Center as part of their clinical routine with serial scans on the same day. The inter-scan interval at Johns Hopkins was assumed to be sufficiently short to prevent any significant plaque volume changes. Patients enrolled at Cedars–Sinai Medical Center underwent their second scan due to image artefacts within one or more segments in the first scan; these were typically misalignment artefacts, not affecting plaque volume acquisitions. Coronary artery segments with degraded image quality were excluded from the scan-rescan analyses of quantitative plaque volumes. None of the participants had undergone previous revascularization.

### Image acquisition and reconstruction

All participants were scanned using a dual-source CT (Johns Hopkins: Siemens Somatom Definition Flash; Cedars–Sinai Medical Center: Siemens Somatom Force) with 280 ms rotation time and 2 × 128 × 0.6 mm collimation.

At Johns Hopkins, participants with a heart rate < 70 beats per minute during image acquisition were scanned at 65–75% of the RR-interval, whereas those with a heart rate of ≥ 70 beats per minute were scanned at 35–75% of the RR-interval. Participants did not receive routine medication. An initial 10 mL test bolus of iodine contrast (Visipaque 320 mgl/mL, GE Medical Systems) was administered with subsequent low-dose image acquisitions to determine the optimal acquisition timing. This was followed by diagnostic imaging with administration of 80–100 mL contrast at a rate of 5–6 mL/s in the antecubital vein. Diagnostic image acquisition was performed with a tube voltage of 100 or 120 kVp, depending on patient size, and with a tube current of 600–800 mA, using Siemens Care Dose 4D software to keep radiation dose at a minimum. Images were reconstructed at 0.75 mm slice thickness with 0.50 mm increments using a B26f reconstruction kernel.

At Cedars–Sinai Medical Center, patients with a heart rate of ≥ 70 beats per minute without contraindications for beta-blockers were administered oral and/or intravenous Metoprolol to achieve a heart rate < 70 beats per minute. Immediately before scanning, 0.4 mg sublingual nitroglycerin spray was administered. An injection of 80–100 mL iodine contrast (Omnipaque 350 mgl/mL, GE Medical Systems) was administered in the antecubital vein, followed by 50 mL of saline chaser. Diagnostic imaging was triggered by real-time bolus tracking with a threshold of 100 HU within an area of interest in the ascending aorta. Scanning was performed by prospective triggering, or helical retrospective gating, at the discretion of the imaging physician based on heart rate and RR-interval consistency with images acquired from 40% to 70% of the cardiac cycle. Tube voltage was 120 kVp and tube current 600 to 800 mA, using ECG-guided dose modulation for spiral acquisitions. Images were reconstructed at 0.50 mm slice thickness with 0.30 mm increments using a BV40 reconstruction kernel.

Repeat scans for all participants were performed according to the same acquisition protocol as their first scan.

### Image analysis

Coronary artery plaque was assessed per-patient across the entire coronary tree in vessel segments with a diameter of at least 2 mm, including side-branches off the main vessels, using AI-enabled validated software (Autoplaque version 3.0). Centerlines were automatically generated following manual definition of each vessel’s ostium and distal end. The centerlines were reviewed by reader 1 in all serial scans to ensure that the readable vessels were assessed uniformly and shared similar vessel volumes across the two scans. The software automatically delineated the vessel wall and lumen, with the difference in volume between these two segmentations constituting the coronary plaque volume. The segmentations were manually adjusted at the discretion of the reader as deemed appropriate to only include true plaque.

To accurately identify differences in plaque volume, the serial scans were analyzed side-by-side on two high-resolution screens, allowing for comparison of the images in a similar manner to what is practiced in clinical imaging laboratories. All serial scans were assessed according to this procedure by two experienced readers trained in the core laboratory at Cedars–Sinai Medical Center. Reader 1 had more than three years of experience in quantitative plaque assessments, and reader 2 had more than 1.5 years of experience in clinical CCTA reporting and quantitative plaque assessments. In case of uncertainty in plaque adjudication, the readers had the option to consult a third experienced reader (more than 8 years of experience) to reach a consensus decision. For statistical analysis, plaque volumes were averaged between the two readers to reduce the impact of individual bias.

Scan-specific thresholds for CP and NCP were software-generated based on contrast enhancement in the ascending aorta and adjusted over the coronary tree [2, 11]. There is no established method for the generation of scan-specific thresholds for LD-NCP, which was defined as plaque of < 30 HU.

Volumes with fixed thresholds for CP and NCP were also exported from the same segmentations with pre-specified thresholds at ≥ 350 HU for CP and −30 to 350 HU for NCP [12].

The default window setting for reader validation of CP quantifications comprised a width of approximately 1500 and a level of approximately 500. Window settings could be altered at the discretion of the readers to guide corrections of the segmentations if needed. A higher window width/level typically reduces calcific blooming, allowing for a more reliable assessment of calcium distribution in the coronary arteries. Segmentations were manually adjusted to avoid the inclusion of excessive calcium blooming. No thresholds were altered. NCP was assessed with a default window width of 465 and a level of 195.

### Statistics

Variable distribution was tested with the Shapiro–Wilk test and inspected in frequency histograms. Approximately normally distributed variables are reported in mean and standard deviation (SD), while skewed variables are reported in median and interquartile range (IQR). Categorical variables are reported as frequency and proportion of the total population (%).

The primary analysis evaluated the reliability of averaged scan-rescan plaque volumes and burden acquisitions per-patient across all subtypes (TP, CP, NCP, LD-NCP). Differences in plaque volumes and burden were tested with the Wilcoxon signed-rank test. Reliability and repeatability were assessed by the intra-class correlation coefficient (ICC [main analysis model: two-way random, type: absolute agreement; within-reader model: two-way mixed]) and the repeatability coefficient (defined as 1.96 × SD of the absolute mean difference). Bland–Altman plots for volumes and burden of TP and plaque sub-components were reported to visualize differences in scan-rescan assessments.

The analyses were performed in SPSS, IBM SPSS Statistics version 28.0.1.0 (142), and MedCalc version 23.2.7. All hypotheses were two-sided and tested at a 5% significance level.

## Results

### Participant characteristics

**Fig. 1:**
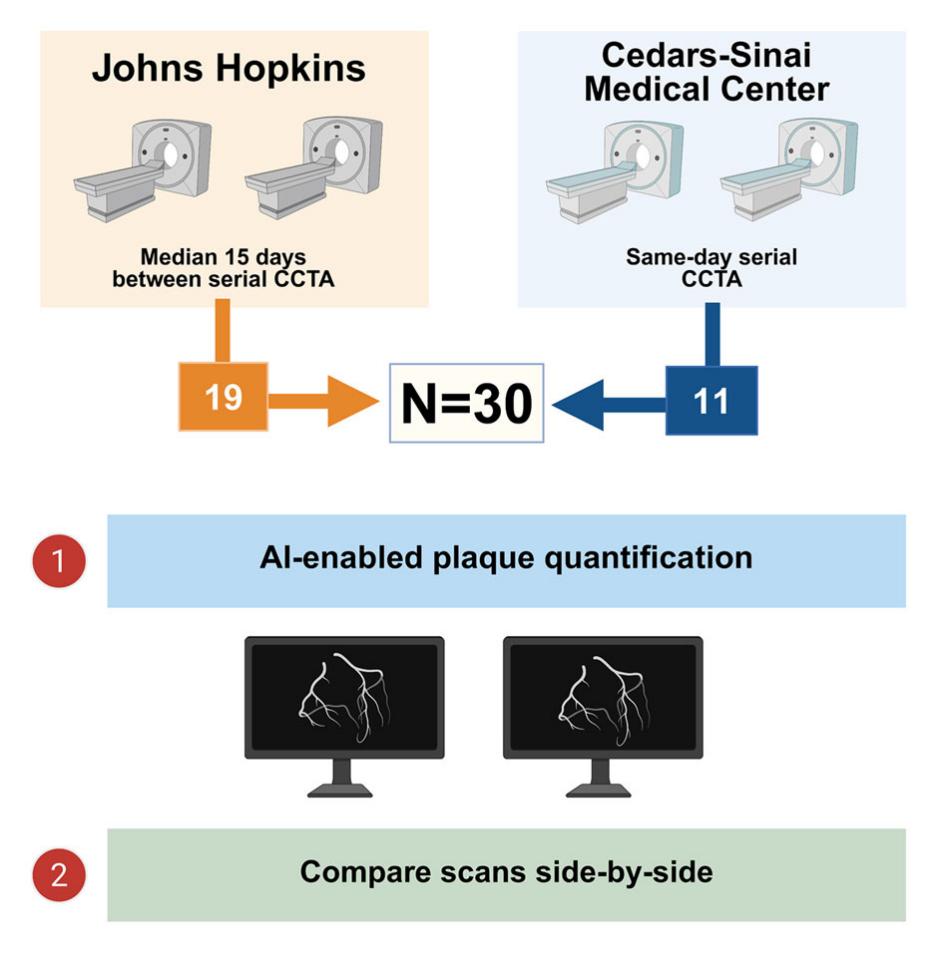
Patient selection and plaque assessment. CCTA, cardiac CT angiography

All 30 participants were included (Fig. 1), the mean age was 59 years, and 70% were men (Table 1). Participants enrolled at Johns Hopkins were younger than those recruited at Cedars–Sinai Medical Center (mean age 52 vs 71 years, *p* < 0.001).

**Table 1:** Baseline characteristics

Characteristics	*N* = 30
Age (years), mean (SD)	59.1 (SD ± 12.6)
Male gender, *n* (%)	21 (70)
Radiation dose (DLP [mGy × cm]), median (IQR)^*^	879 (644–1002)
Clinical history, *n* (%)	
Hypertension	18 (60)
Hypercholesterolemia	18 (60)
Diabetes	6 (20)
Stroke	1 (3)
History of smoking	16 (53)

* DLP reported as average of the serial scans per patient

*DLP* Dose Length Product, *IQR* Interquartile range

The entire RCA was excluded in three participants, and the proximal segment of the LAD was excluded in one participant due to motion artefacts in the first scan, preventing reliable adjudication of plaque. All remaining eligible segments of a diameter ≥ 2 mm within all participants were of sufficient image quality for plaque quantification in both scans. No participants had undergone previous angioplasty or bypass grafting. A majority (60%) had known hypercholesterolemia and hypertension.

Plaque volume and burden quantifications are reported in Table 2. Median TP volume in scan 1 was 159 mm^3^ (IQR 81–302) vs 160 mm^3^ (IQR 79–314) in scan 2, *p* = 0.405, with a dominance of NCP (Scan 1: 88 mm^3^ [IQR 42–143] vs Scan 2: 106 mm^3^ [38–159], *p* = 0.465). TP burden was 19% (IQR 12–24) in scan 1 vs 18% (IQR 12–24) in scan 2, *p* = 0.658, with a median NCP burden at 12% (IQR 4–19) vs 10 (IQR 5–19), *p* = 0.478, in scan 1 and 2, respectively.

**Table 2:** Scans 1 and 2 comparison of plaque volumes and burden derived from scan-specific thresholds

	Scan 1	Scan 2	*p*-value
Plaque volume, mm^3^			
TP	159 (81–302)	160 (79–314)	0.405
CP	35 (15–153)	32 (13–155)	0.982
NCP	88 (42–143)	106 (38–150)	0.465
LD-NCP	4 (1–6)	2 (1–6)	0.045
Plaque burden, %			
TP	19 (12–24)	18 (12–24)	0.658
CP	4 (2–10)	4 (2–10)	0.964
NCP	12 (4–19)	10 (5–19)	0.478
LD-NCP	0.2 (0.1–0.5)	0.2 (0.1–0.5)	0.170

*CP* calcified plaque, *LD-NCP* low-density non-calcified plaque, *NCP* non-calcified plaque, *TP* total plaque

Patients from the participating sites differed in age and plaque characteristics, with those enrolled at Cedars–Sinai Medical Center, being scanned as part of clinical practice, demonstrating higher volumes of CP and lower volumes of NCP compared to participants from Johns Hopkins (Supplemental Table 1).

### Scan-rescan differences in plaque

There were no statistically significant differences in scan-rescan averaged volumes for TP, CP, and NCP, while LD-NCP volume differed with statistical significance (4 mm^3^ [1–6] vs 2 mm^3^ [0–6], *p* = 0.045) between the scans.

**Fig. 2:**
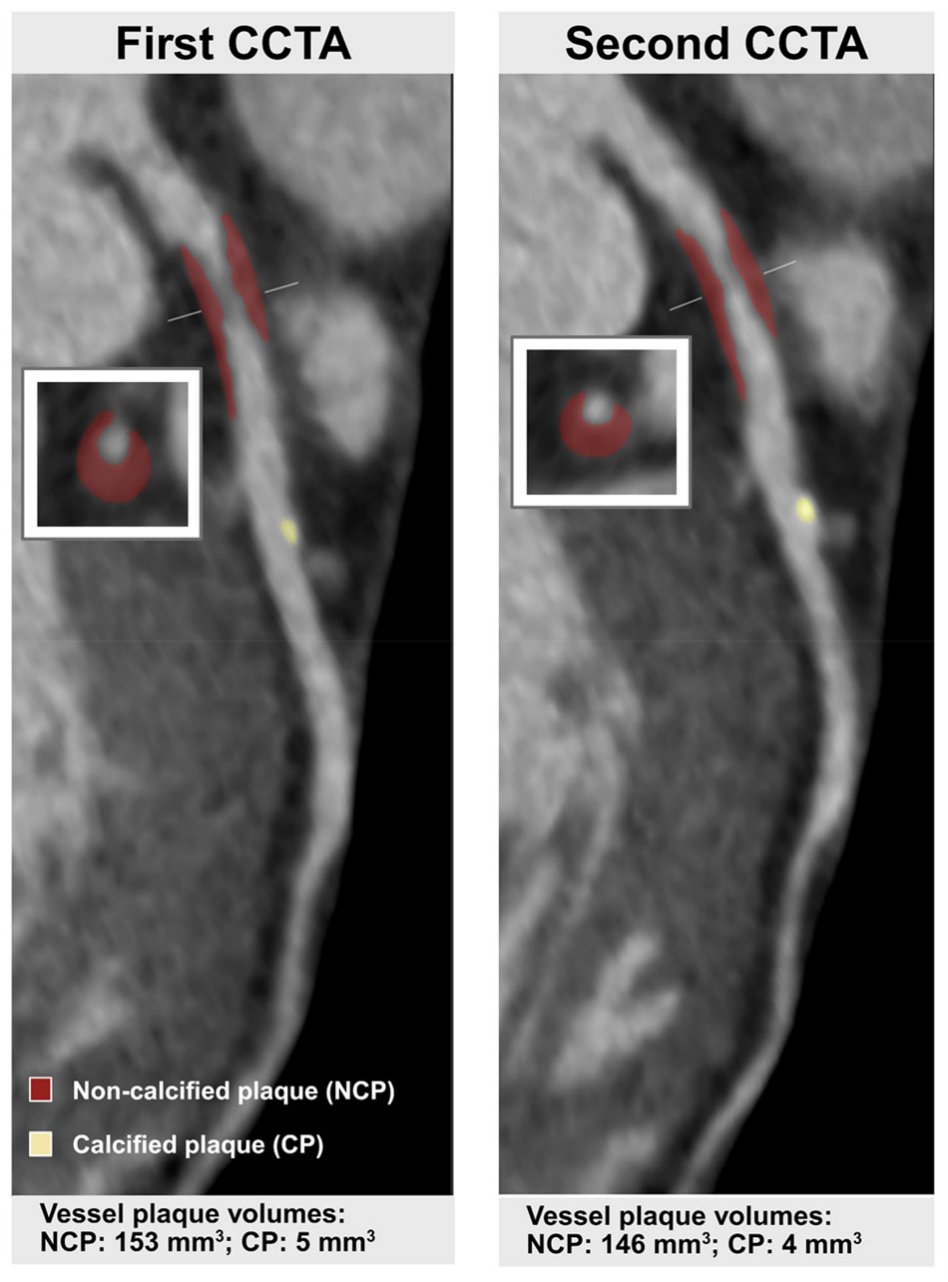
Example of plaque adjudication side-by-side in one vessel. CCTA, cardiac CT angiography

Individual reader acquisitions are reported in Supplemental Table 2. Reader 2 reported a small but statistically significant scan-rescan difference in LD-NCP volume (3 mm^3^ [1–6] vs 2 [0–5], *p* = 0.031), TP burden (20% [IQR 13–29] vs 17% [IQR 12–28], *p* = 0.033) and NCP burden (14% [IQR 5–22] vs 11% [IQR 4–22], *p* = 0.047) while reader 1 reported no statistically significant differences in any plaque metric. An example-image of serial acquisitions in one vessel is presented in Fig. 2.

### Reliability and reproducibility

**Fig. 3:**
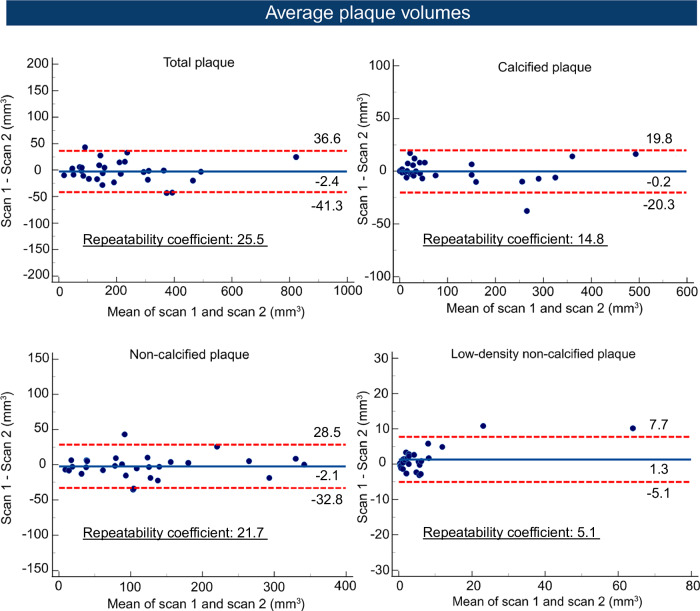
Bland–Altman plots of scan-rescan differences in plaque volumes derived from scan-specific thresholds

Metrics of reproducibility for plaque volumes and burden are reported in Table 3 and individual reader data are reported in Supplemental Table 3. Overall, the ICC was excellent for scan-rescan volume assessments across all plaque components. LD-NCP volume presented with the lowest ICC at 0.98 (95% CI: 0.95–0.99). ICC for TP was 1.00 (95% CI: 0.99–1.00). Plaque burden was also assessed with excellent ICC, showing a similar pattern with the lowest values for LD-NCP burden at 0.96 (95% CI: 0.91–0.98), while TP burden had an ICC of 0.98 (95% CI: 0.96–0.99). Scan-rescan differences of plaque volumes are depicted in Bland–Altman plots shown in Fig. 3, while Supplemental Fig. 1 shows the Bland–Altman plots for differences in plaque burdens. Supplemental Fig. 2 shows Bland–Altman plots for inter-rater agreement of volume quantifications.

**Table 3:** Metrics of scan-rescan reliability (mean absolute difference, repeatability coefficient, and Bland–Altman limits of agreement

	Mean difference	Intra-class correlation coefficient	Repeatability coefficient	Limits of agreement
Plaque volume				
TP	15.0 mm^3^	1.00 (95% CI: 0.99–1.00)	25.5	−41.3 to 36.6
CP	6.8 mm^3^	1.00 (95% CI: 1.00–1.00	14.8	−20.3 to 19.8
NCP	11.1 mm^3^	0.99 (95% CI: 0.99–1.00)	21.7	−32.8 to 28.5
LD-NCP	2.3 mm^3^	0.98 (95% CI: 0.95–0.99)	5.1	−5.1 to 7.7
Plaque burden				
TP	1.5%	0.98 (95% CI: 0.96–0.99)	2.6	−4.1 to 3.8
CP	0.6%	0.99 (95% CI: 0.99–1.00)	1.3	−1.7 to 1.8
NCP	1.2%	0.99 (95% CI: 0.98–1.00)	2.4	−3.6 to 3.3
LD-NCP	0.3%	0.96 (95% CI: 0.91–0.98)	0.8	−0.7 to 1.0

*CP* calcified plaque, *LD-NCP* low-density non-calcified plaque, *NCP* non-calcified plaque, *TP* total plaque

The inherent scan-rescan variability, as determined by the repeatability coefficient, for TP volume was 25.5 mm^3^ and 2.6% for TP burden. CP was assessed with greater consistency than NCP. The inherent variation in CP and NCP volume was 14.8 mm^3^ and 21.7 mm^3^. Corresponding values for CP burden and NCP burden were 1.3% and 2.4%, respectively. The repeatability coefficient for LD-NCP volume was 5.9 mm^3^ and for LD-NCP burden 1.5%. CP and NCP volume indexed by TP volume, typically termed CP or NCP composition, had a repeatability coefficient at 6.7%.

**Fig. 4:**
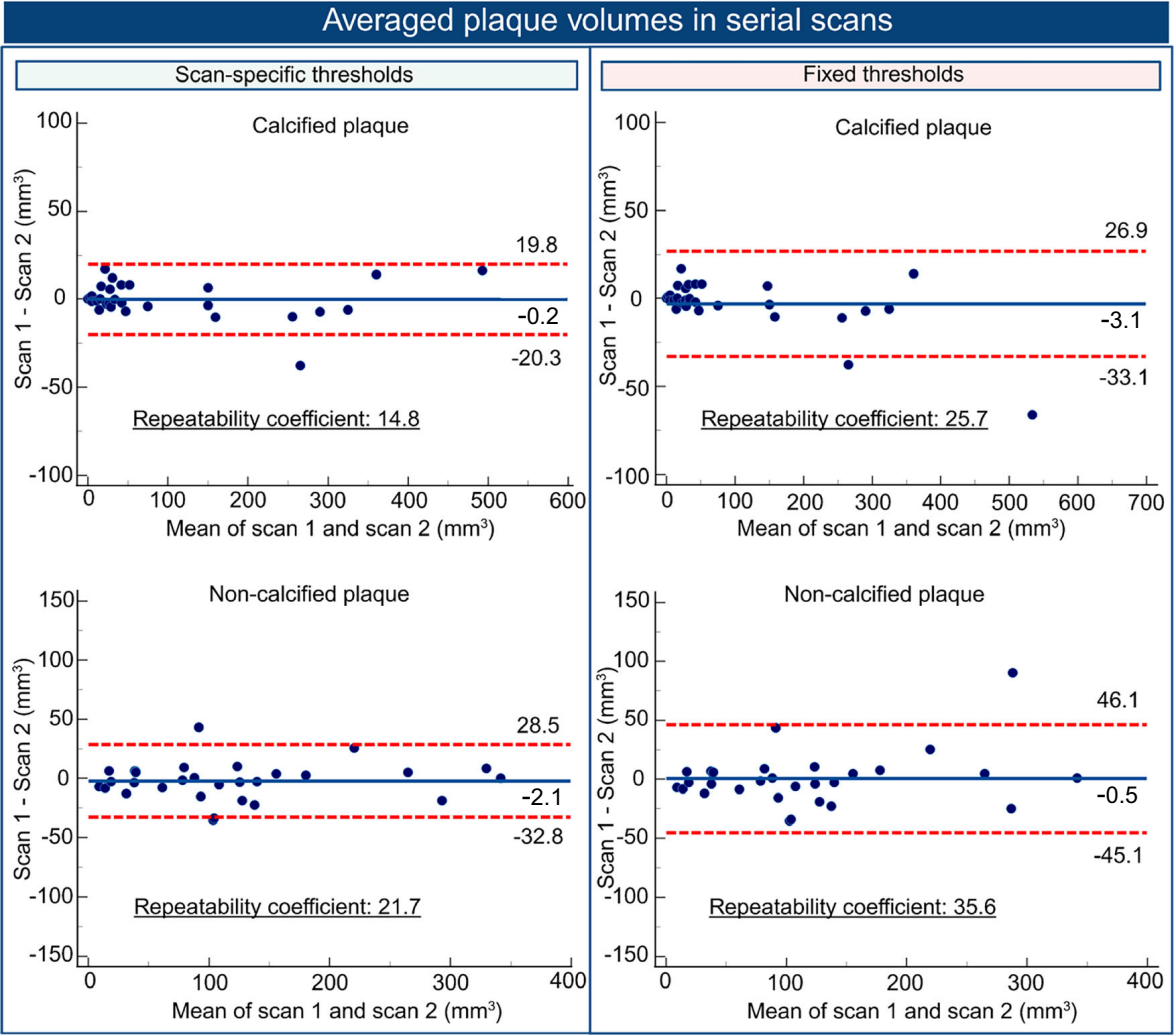
Repeatability with scan-specific thresholds vs fixed thresholds

Figure 4 shows Bland–Altman plots for CP and NCP assessed using scan-specific and fixed thresholds. Although mean differences are similar, fixed thresholds demonstrate a greater variation as depicted by wider limits of agreement (CP: −20.3 to 19.8 vs −33.1 to 26.9; NCP: −32.8 to 28.5 vs −45.1 to 46.1, for scan-specific and fixed thresholds, respectively). This wider variability for fixed thresholds is also reflected in higher repeatability coefficients.

## Discussion

This paper presents a framework for AI-enabled assessments of quantitative plaque in serial CCTA scans with high reproducibility and reliability. This approach may be feasible for adoption in clinical practice among selected patients with good image quality. We further present thresholds, determined by the repeatability coefficient, that may be adopted to differentiate true biological changes in plaque volumes and burden from inherent variations in the acquisition method.

In our study, we have leveraged the latest generation of AI-enabled plaque quantification software in a side-by-side analysis of repeated scans, demonstrating improved reproducibility compared to previous reports. The side-by-side approach to maximize segmentation consistency and diagnostic reliability is widely accepted across different modalities in medical imaging and was recently suggested as a method to reduce variability of serial CCTA scans in a society expert consensus paper [10, 13, 14]. This study is, to our knowledge, the first to evaluate the reliability of the side-by-side approach in CCTA plaque quantification. It is also the first study to report reliability and repeatability metrics for plaque volumes from both scan-specific and fixed thresholds applied on the same segmentations.

Coronary plaque composition offers incremental prognostic information beyond clinical data and CAD burden, emphasizing the importance of reliable assessments [15]. Reproducibility of serial coronary plaque volume quantifications from CCTA are also of utmost importance, as CCTA is increasingly leveraged to objectively follow CAD progression over time, as exemplified by a recent study demonstrating the association between higher Lp(a) concentrations and progression of low-attenuation plaque in diabetic patients [16]. Serial scans should preferably be performed on the same scanners, and image acquisition protocols should be consistent, as changes may impact the quantification of plaque volumes [17]. Lower tube voltage is known to increase the attenuation of the iodine contrast within the blood pool, which is associated with higher volumes of quantified CP [18]. This overestimation of CP may in part be attributed to increased calcium blooming at lower tube voltages, but it is also known that NCP attenuation varies with luminal attenuation and acquisition parameters [10, 19, 20]. Furthermore, patient-specific factors, such as body size, also affect luminal attenuation in coronary CTA.

In this study, different contrast media with slightly differing iodine density were used at the participating sites, which may have affected the quantified absolute plaque volumes, however, given that acquisition protocols were consistent across scans for individual participants, this most likely did not affect reproducibility. The interaction between lumen opacification and plaque volumes may be circumvented by the use of scan-specific plaque thresholds adjusted for blood pool attenuation to achieve reliable plaque quantifications, although this feature is not available in all software packages [11].

We show that scan-specific thresholds for quantification of CP and NCP offer higher consistency than fixed thresholds. By observing the Bland–Altman plots, it is clear that the higher repeatability coefficients, and wider limits of agreement, with fixed thresholds, were driven by outliers contributing to an increase in SD. Although fixed thresholds appeared to offer reliable quantifications of plaque in most cases, this susceptibility to produce divergent volumes in a minority of scans would favor the adoption of scan-specific thresholds when high reproducibility is a priority.

LD-NCP was the only plaque component to differ with statistical significance between the serial scans. Despite this tendency of lower reproducibility, LD-NCP has been reported to be of a particularly high prognostic value [4]. This is the only plaque component not assessed with scan-specific thresholds, which may explain this greater variation.

The high reproducibility in our study was achieved at a core lab by readers with extensive experience in quantitative plaque analysis using AI-enabled, validated plaque software [21, 22]. It has previously been reported that different vendors yield different volumes of plaque, emphasizing the importance of performing scan-rescan measurements with the same software by an operator who is trained on the specific platform [23]. It is generally practiced across vendors to define plaque volume as the difference between the segmented volume delineated by the vessel wall and the segmented volume of the vessel lumen [10]. Hence, the workflow to quantify plaque using different platforms is overall uniform, mainly differing depending on the extent of AI-facilitated steps. A strength of the software used in this study is the option to derive scan-specific thresholds that evidently are more reproducible than the fixed thresholds offered by some of the other vendors [10, 24]. It should be noted that the scan-specific thresholds not only offer better reproducibility; they have also been validated against IVUS, demonstrating robustness across different vendor packages [21, 25].

Previous studies examining reproducibility of quantitative scan-rescan plaque metrics reported high coefficients of repeatability and wide limits of agreement, particularly in assessments of NCP volume [8, 26, 27]. These studies all applied blinded assessments of plaque volumes, revealing challenges in the quantification of plaque changes with high precision when the baseline scan is not available. In contrast, our study demonstrates that systematic side-by-side analyses offer consistency in analyses of serial scans. This is in part achieved by ensuring that the serial acquisitions share the same vessel length and that the segments with non-diagnostic image quality are excluded from both scans.

### Limitations

This study has limitations to address. First, the sample size is limited, which prevents reliable assessments of variations in plaque metrics across a wider range of plaque volumes. This limitation is related to the scarcity of existing serial contrast studies within a short time range, as demonstrated by the need for pooled data from two institutions to achieve this sample size of 30 participants.

This study demonstrates high reproducibility of serial plaque quantifications using side-by-side readings, but there is no comparator arm, which prevents any assessment of incremental benefit from this approach compared to other methods. We do, however, demonstrate the superior reproducibility of scan-specific thresholds compared to fixed thresholds.

It should also be noted that the side-by-side plaque quantification approach is more time-consuming than solely quantifying plaque in the latest scan and comparing the findings to the previous scan report. However, the side-by-side practice is more in line with clinical practice, with AI-enabled plaque analysis allowing an acceptable workflow [22].

This study investigated the reproducibility of plaque volumes across serial acquisitions on the same scanner using consistent scanning protocols. In real-world practice, this consistency may be difficult to achieve as older scanners are upgraded over time and patients change their geographical location, leading to serial scans at different sites with varying hardware and protocols. Hence, future scientific efforts should aim to improve the reproducibility of plaque volumes also across inconsistent scanning modes.

## Conclusion

Coronary plaque volume and burden were quantified in serial CCTA scans with high reproducibility and reliability across all subcomponents using a systematic assessment method and scan-specific attenuation thresholds with AI-enabled plaque software. This study outlines a workflow to maximize consistency in scan-rescan measures and provides reference values to determine a true change in coronary plaque from inherent variations in the acquisition method.

## Supplementary information

Supplementary information

## Abbreviations

AI: Artificial intelligence
CAD: Coronary artery disease
CCTA: Cardiac computed tomography angiography
CP: Calcified plaque
LD-NCP: Low-density non-calcified plaque
NCP: Non-calcified plaque
TP: Total plaque
