# Supplementary material for: High scan-rescan repeatability of AI-enabled coronary plaque quantification from coronary CT angiography
Source: Eur Radiol. 2026 May 12;36(9):7129–38. doi: 10.1007/s00330-026-12598-1 (PMC13451515; doi:10.1007/s00330-026-12598-1)

High Scan-Rescan Repeatability of AI-enabled coronary plaque quantification from Coronary CT Angiography

ELECTRONIC SUPPLEMENTARY MATERIAL

Supplemental Table 1. Inter-site differences in plaque volumes and absolute delta between the first and second scan using scan-specific thresholds:

	Johns Hopkins (N=19)	CSMC (N=11)	P-value
Scan 1, total plaque, mm3	158.2 (113.0-253.2)	179 (46.9-363.6)	0.846
Scan 1, calcified plaque, mm3	29.7 (10.3-56.0)	154.2 (26.5-286.6)	**0.030**
Scan 1, non-calcified plaque, mm3	126.4 (85.8-233.0)	42.7 (20.4-85.5)	**<0.001**
Scan 1, low-density non-calcified plaque, mm3	4.1 (0.8-9.1)	2.8 (0.4-4.6)	0.189
∆Total plaque, mm3	14.7 (4.8-27.8)	9.5 (3.4-23.0)	0.846
∆Calcified plaque, mm3	4.0 (0.9-7.4)	7.0 (2.8-10.2)	0.127
∆Non-calcified plaque, mm3	6.2 (2.7-18.5)	6.8 (5.3-12.9)	0.401
∆Low-density non-calcified plaque, mm3	2.3 (0.9-3.3)	1.2 (0.2-2.3)	**0.037**

Reported median and interquartile ranges.

Supplemental Table 2. Plaque metrics per reader

**Reader 1**	**Scan 1**	**Scan 2**	**P-value**
**Plaque volume, mm^3**			
Total plaque	155 (78-324)	159 (87-318)	0.072
Calcified plaque	36 (15-163)	31 (14-168)	0.374
Non-calcified plaque	89 (38-145)	110 (45-148)	0.066
Low-density non-calcified plaque	3 (1-7)	2 (1-8)	0.213
**Plaque burden, %**			
Total plaque	19 (13-27)	19 (13-28)	0.530
Calcified plaque	5 (2-11)	5 (2-10)	0.194
Non-calcified plaque	13 (4-22)	11 (5-22)	0.262
Low-density non-calcified plaque	0.2 (0.1-0.6)	0.3 (0.1-0.6)	0.504

**Reader 2**	**Scan 1**	**Scan 2**	**P-value**
**Plaque volume, mm^3**			
Total plaque	161 (93-295)	171 (83-310)	0.329
Calcified plaque	34 (15-145)	32 (15-156)	0.665
Non-calcified plaque	98 (44-152)	95 (35-161)	0.360
Low-density non-calcified plaque	3 (1-6)	2 (0-5)	**0.031**
**Plaque burden, %**			
Total plaque	20 (13-29)	17 (12-28)	**0.033**
Calcified plaque	5 (2-10)	5 (2-9)	0.059
Non-calcified plaque	14 (5-22)	11 (4-22)	**0.047**
Low-density non-calcified plaque	0.2 (0.1-0.5)	0.2 (0.1-0.4)	0.069

Supplemental Table 3. Metrics of scan-rescan reliability per reader

Reader 1	Mean difference	Intra-class correlation coefficient	Repeatability coefficient	Limits of Agreement
**Plaque volume**				
Total plaque	20.3 mm^3	1.00 (95% CI 0.99-1.00)	30.5	-56.2 to 39.6
Calcified plaque	8.1 mm^3	1.00 (95% CI 1.00-1.00	17.8	-25.6 to 21.8
NCP	16.8 mm^3	0.99 (95% CI 0.97-0.99)	27.2	-47.7 to 34.8
LD-NCP	2.5 mm^3	0.97 (95% CI 0.94-0.99)	5.9	-6.3 to 8.5
**Plaque burden**				
Total plaque	2.8 %	0.96 (95% CI 0.92-0.98)	5.2	-7.9 to 7.5
Calcified plaque	0.7 %	0.99 (95% CI 0.98-1.00)	1.3	-1.6 to 2.1
NCP	2.5 %	0.98 (95% CI 0.95-0.99)	4.6	-7.2 to 6.3
LD-NCP	0.4 %	0.96 (95% CI 0.92-0.98)	1.2	-1.2 to 1.5
**Reader 2**	**Mean difference**	**Intra-class correlation coefficient**	**Repeatability coefficient**	**Limits of Agreement**
**Plaque volume**				
Total plaque	17.4 mm^3	1.00 (95% CI 0.99-1.00)	26.3	-39.3 to 46.5
Calcified plaque	7.1 mm^3	1.00 (95% CI 1.00-1.00)	19.2	-22.3 to 25.1
NCP	13.3 mm^3	0.99 (95% CI 0.98-1.00)	18.7	-29.9 to 34.3
LD-NCP	2.6 mm^3	0.97 (95% CI 0.94-0.99)	5.6	-5.4 to 8.5
**Plaque burden**				
Total plaque	2.5 %	0.93 (95% CI 0.85-0.97)	4.1	-4.9 to 7.2
Calcified plaque	0.9 %	0.99 (95% CI 0.98-1.00)	1.8	-1.9 to 2.8
NCP	1.8 %	0.95 (95% CI 0.89-0.98)	3.6	-4.2 to 5.5
LD-NCP	0.4 %	0.88 (95% CI 0.73-0.94)	1.5	-1.4 to 1.9

Supplemental Figure 1. Bland-Altman plots of scan-rescan differences in plaque burden

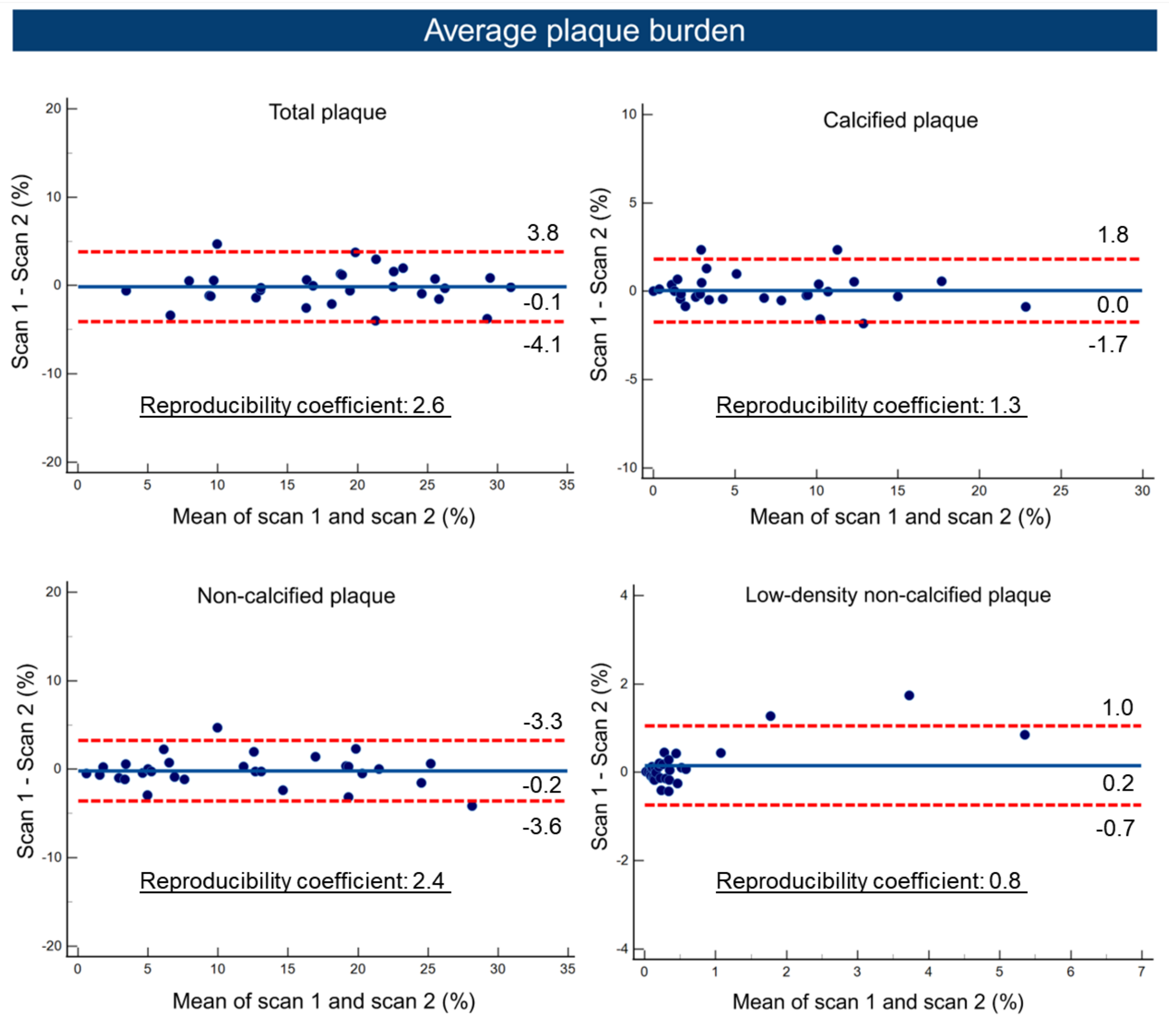

Supplemental Figure 2: Bland-Altman plot of plaque volume assessments by reader 1 and 2.

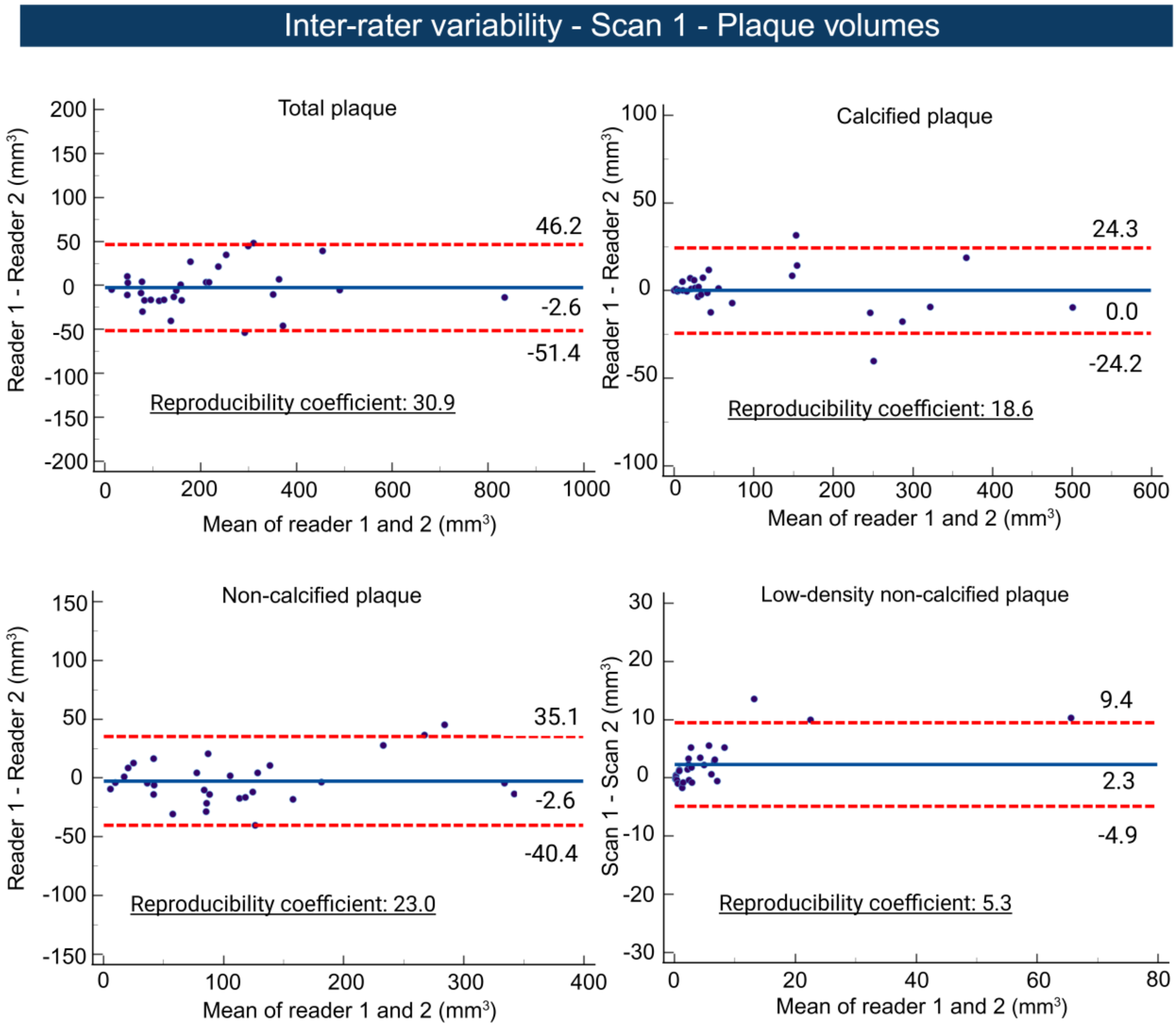

Supplement: Supplementary file 1 [file 330_2026_12598_MOESM1_ESM.pdf]
